# Metabolic regulation of apoptosis via Ca+/Calmodulin Kinase II (CaMKII)

**DOI:** 10.1186/1753-6561-6-S3-P36

**Published:** 2012-06-01

**Authors:** Francis McCoy, Laura Eckard, Si-lng Chen, Leta K Nutt

**Affiliations:** 1 Biochemistry Department, St. Jude Children’s Research Hospital, Memphis, Tennessee, USA

## Background

The metabolic state of a cell is an important factor in whether or not it engages its apoptotic machinery. Furthermore, reprogramming energy metabolism is now recognized as a hallmark of cancer; so understanding how metabolism regulates apoptosis is crucial. Recently it has been demonstrated that CaMKII serves as a link between metabolic state and apoptosis [1]. Using the *Xenopus laevis* oocyte extract, it was shown that addition of glucose-6-phosphate (G6P) induced an inhibitory phosphorylation of caspase 2, mediated via CaMKII. However, the mechanisms of how G6P and metabolism regulate CaMKII still remain unclear. The aim of this study is to investigate the underlying mechanisms of how metabolism regulates CaMKII activity.

## Materials and methods

*Preparing Xenopus oocyte extracts* Mature female frogs were induced to lay eggs, and crude extracts prepared as described previously [1].

*Metabolomics* Metabolomics analysis of cytosol fractionated from extracts treated with, or without G6P was performed by GC/MS and LC/ MS/MS (Metabolon).

*Kinase Assays* Recombinant GST pro-caspase 2 fusion proteins were incubated in crude extract with 5 µCi [γ^32^P]ATP and metabolites for 1.5hr at room temperature, and retrieved on glutathione sepharose beads.

## Results

Following metabolomic analysis, we found increased acetyl CoA levels in cytosol fractionated form G6P treated extracts. Although increased cytosolic acetyl CoA increased global acetylation in the extract, we found no evidence of caspase 2 or CaMKII acetylation. We found that treatment of extract with acetyl CoA was capable of inhibiting apoptosis at the level of caspase 2 and caspase 3. Furthermore, acetyl CoA induced phosphorylation of CaMKII and subsequent phosphorylation of caspase 2. Acetyl CoA is an important pre-cursor for fatty acid and cholesterol synthesis. However, the products of these pathways, palmitate and mevalonic acid respectively, had no effect on CaMKII or apoptosis. Furthermore, immuno-depletion of FAS had no effect on the ability of acetyl CoA to inhibit apoptosis or activate CaMKII. Our metabolomics also showed no significant difference in the products of these pathways.

## Conclusions

Using an unbiased metabolomics approach, and a targeted approach looking at specific pathways, we have found that increased acetyl CoA following G6P treatment signals CaMKII activation in an acetylation-independent manner. Inhibition of CaMKII has been shown to be an effective complimentary strategy in treating neuroblastomas [2]. Furthermore, CaMKII has also been shown to be an important regulator of apoptosis in prostate cancer cells [3]. Therefore, determining how metabolism regulates CaMKII may allow us to better understand how metabolism promotes cancer cell survival. We hypothesize that acetyl CoA, and possibly other short chain acyl CoAs, may function as a cofactor for a cytosolic enzymatic process involved in activating CaMKII. We are currently testing this hypothesis in the lab at present.
